# How Do Internal and External CSR Affect Employees' Organizational Identification? A Perspective from the Group Engagement Model

**DOI:** 10.3389/fpsyg.2016.00788

**Published:** 2016-05-30

**Authors:** Imran Hameed, Zahid Riaz, Ghulam A. Arain, Omer Farooq

**Affiliations:** ^1^Lahore Business School, The University of LahoreLahore, Pakistan; ^2^Faculty of Business Administration, Lahore School of EconomicsLahore, Pakistan; ^3^Department of Human Resources, Effat College of Business, Effat UniversityJeddah, Saudi Arabia; ^4^Kedge Business SchoolMarseille, France

**Keywords:** CSR, organizational identification, group engagement model, respect, prestige

## Abstract

The literature examines the impact of firms' corporate social responsibility (CSR) activities on employees' organizational identification without considering that such activities tend to have different targets. This study explores how perceived *external* CSR (efforts directed toward external stakeholders) and perceived *internal* CSR (efforts directed toward employees) activities influence employees' organizational identification. In so doing, it examines the alternative underlying mechanisms through which perceived external and internal CSR activities build employees' identification. Applying the taxonomy prescribed by the group engagement model, the study argues that the effects of perceived external and internal CSR flow through two competing mechanisms: perceived external prestige and perceived internal respect, respectively. Further, it is suggested that calling orientation (how employees see their work contributions) moderates the effects induced by these alternative forms of CSR. The model draws on survey data collected from a sample of 414 employees across five large multinationals in Pakistan. The results obtained using structural equation modeling support these hypotheses, reinforcing the notion that internal and external CSR operate through different mediating mechanisms and more interestingly employees' calling orientation moderates these relationships to a significant degree. Theoretical contributions and practical implications of results are discussed in detail.

## Introduction

The widespread growth of corporate social responsibility (CSR) practices makes it important to determine how they influence different stakeholders such as employees, consumers, investors, suppliers, and the government (Aguinis and Glavas, 2012). Among these groups, employees are vital to any discussion of the origins and consequences of CSR (Aguilera et al., 2007). However, most micro CSR research focuses on external stakeholders such as consumers and investors (Lichtenstein et al., 2004; Luo and Bhattacharya, 2006; Sen et al., 2006), neglecting employees as a key and integral stakeholder group (Larson et al., 2008). Although some recent studies assess the impact of firms' CSR activities on employees' attitudes and behaviors (e.g., Brammer et al., 2007; Turker, 2009a; Mueller et al., 2012; Zhu et al., 2012), most of these studies have focused on the direct relationship between the two rather than on the underlying mechanisms and boundary conditions through which CSR influences employee outcomes. This study attempts to address this gap.

Most micro CSR studies focus on organizational identification as a significant CSR outcome because it is a fundamental construct that predicts relevant behaviors (Albert et al., 2000). For instance, Collier and Esteban (2007), Farooq M. et al. (2014); Farooq O. et al. (2014), and Rodrigo and Arenas (2008) demonstrate the positive relationship between CSR and organizational identification. While these studies contribute greatly to our understanding of how CSR affects employees' identification, most of them suggest there is a direct link between CSR and organizational identification. Kim et al. (2010), Jones (2010), and De Roeck and Delobbe (2012) demonstrate this link through the mediation of perceived prestige or pride. Conversely, they do not consider whether a number of underlying mechanisms induced by different types of CSR activities influence organizational identification.

We argue that CSR generates a number of mediators that influence organizational identification; this is because CSR comprises a variety of discretionary actions taken by the firm, targeting different stakeholder groups. To better understand how different kinds of CSR influence employees' identification, we differentiate between *internal* and *external* CSR activities (Cornelius et al., 2008; Jones and Rupp, 2016) in this context and suggest different underlying mechanisms through which CSR fosters identification.

Scholars have suggested that the impact of CSR on employee outcomes is sensitive to how an individual is oriented (e.g., Rupp et al., 2013a,b; Bridoux et al., 2016). Particularly, researchers propose that employees' perception about CSR and its subsequent outcomes are sensitive to their calling orientation (e.g., Glavas and Godwin, 2013), that is, the extent to which employees see their work as a “calling” rather than merely a “job.” Thus, the study also explores how employees' calling orientation can strengthen or weaken the process by which perceived internal and external CSR lead to employees' organizational identification.

We examine how weak and strong calling orientations moderate the effects of perceived external and internal CSR on identification via perceived external prestige and perceived internal respect, respectively. Using social identity theory, we propose that CSR actions focusing on external stakeholders enhance perceived external prestige whereas those focusing on employees increase perceived internal respect. Perceived external prestige and perceived internal respect encourage employees to identify with their socially responsible organization. Furthermore, we suggest that the use of mechanisms based on perceived external prestige and/or perceived internal respect to build organizational identification depend, in turn, on employees' calling orientation.

Finally, given that the bulk of CSR research concentrates on developed countries (Aguinis and Glavas, 2012; Jones et al., 2014), this study shifts the focus by presenting data from South Asia, a developing region-Pakistan. This is in response to scholars who have called for CSR research on other regions of the world (Rupp et al., 2013a). Accordingly, our model relies on self-reported data from a sample of 414 employees working across five large multinationals in Pakistan.

The study contributes to the literature in several ways. First, it examines how perceived internal and external CSR actions influence employees' organizational identification. In so doing, the study shows how alternative underlying mechanisms—perceived external prestige and perceived internal respect—connect components of CSR and organizational identification. This study responds to Aguinis and Glavas (2012), who have emphasized the need to understand such mechanisms in relation to employee outcomes. Exploring these alternative mediation mechanisms could also help strengthen firms' capacity for managing the impact of CSR initiatives (Farooq O. et al., 2014).

Second, the study contributes to both theory and practice by closely gauging how employees perceive and react to CSR. There is no “best way” of carrying out CSR and the difference in employees' calling orientation plays an important role in evaluating such activities. This implies that managers must take into account the differential impact of CSR components on employees in order to design effective CSR strategies. Finally, in suggesting that this impact depends on employees' calling orientation, this study shows how calling orientation acts as a boundary condition of the relationship between perceived CSR and its outcomes (Colquitt and George, 2011).

## Conceptual background and hypotheses

CSR is a set of firm's initiatives that go beyond the notion of profit-making or compliance with the law (McGuire, 1963; Davis, 1973; McWilliams et al., 2006; Aguilera et al., 2007; De Roeck et al., 2014). It entails promoting good causes, instituting good practices, and carrying out philanthropy, all of which highlight a firm's ethical position (Carroll, 1979; Kotler and Lee, 2005). These elements are vital to building a more productive relationship with the firm's stakeholders (Waddock and Smith, 2000; Bhattacharya et al., 2009).

Scholars distinguish between a firm's social initiatives in terms of internal CSR and external CSR, which are directed at internal and external stakeholders, respectively (Verdeyen et al., 2004; Werther and Chandler, 2010; El Akremi et al., 2015). Internal CSR denotes the policy and practices of an organization that are related to the psychological and physiological well-being of its employees (Verdeyen et al., 2004; Brammer et al., 2007; Turker, 2009b; Shen and Jiuhua Zhu, 2011). These include respect for human rights, employee health and safety, work-life balance, employee training, equal opportunity, and diversity (Vuontisjärvi, 2006; Turker, 2009a; Gond et al., 2011; Shen and Jiuhua Zhu, 2011). External CSR relates to environmental and social practices that help to strengthen the firm's legitimacy and reputation among its external stakeholders (Carroll, 1979; Brammer et al., 2007). External CSR activities include volunteerism, cause-related marketing, corporate philanthropy, and environmental and wildlife protection (Brammer et al., 2007; Chen et al., 2008; Cornelius et al., 2008).

Although the literature differentiates between internal and external CSR, most micro CSR research examining the impact of CSR on employees' attitudes and behaviors has rarely tested the differential effects and underlying mechanisms associated with these two types of CSR practices (e.g., Brammer et al., 2007; Mueller et al., 2012; Zhu et al., 2012). This distinction is important from an employee perspective because perceived internal CSR appears to be self-focused whereas perceived external CSR appears to be others-focused. These two facets of CSR can, therefore, affect employees' related attitudes and behaviors differently. This study explores how internal and external CSR produce different pathways through which CSR builds identification.

Recent studies in micro CSR also suggest that CSR affects employees' organizational identification (Glavas and Godwin, 2013, e.g., Farooq M. et al., 2014; Farooq O. et al., 2014; El Akremi et al., 2015). Employees associate themselves strongly with their organization when it is involved in social welfare activities (Smidts et al., 2001; Glavas and Godwin, 2013). Jones (2010) finds that employees who received support from their organization when carrying out community services were more likely to feel strongly attached to the organization. Jones et al. (2014) argue that firms engaged in social wellbeing activities earn a positive image and are better able to attract suitable employees. In a field experiment on CSR and stakeholder relationships, Sen et al. (2006) find that both external as well as internal stakeholders identify better with an organization once they become aware of its CSR activities. However, few studies have looked at how and why specific CSR practices influence identification, this study attempts to fill this gap.

Organizational identification is a specific form of social identification derived from social identity theory (Tajfel and Turner, 1985; Ashforth and Mael, 1989) and self-categorization theory (Haslam and Ellemers, 2005). It is conceptualized as “a perceived oneness with an organization and the experience of the organization's successes and failures as one's own” (Mael and Ashforth, 1992, p. 103). The research indicates that organizational identification is an important determinant of a firm's overall effectiveness (e.g., Pratt, 1998). It has a positive impact on several organizational outcomes such as job satisfaction (Van Dick et al., 2004a), organizational citizenship behavior (Bartel, 2001; Tyler and Blader, 2003) and readiness for change (Hameed et al., 2013), and is negatively related to turnover intention (Mael and Ashforth, 1995). According to Ellemers et al. (2003) and Van Dick et al. (2004b), social identity theory makes the following key assumptions: (i) individuals strive to achieve positive self-esteem, (ii) some part of individuals' self-esteem is based on their social identity derived from group membership, and (iii) in order to evaluate and maintain a positive social identity, a group comparison is required with the relevant out-groups. In order for social identity theory assumptions to be applicable, a minimum level of group identification should exist along with salient membership of the group (Van Dick, 2001).

Social identification also assumes that a person's self-concept consists of two components: his or her own identity and a large number of social identities (Abrams and Hogg, 1988). In other words, it refers to the process by which individuals categorize themselves into several social groups to reinforce their self-esteem and self-concept (Tajfel and Turner, 1986; Hogg and Terry, 2000; Terry and Hogg, 2001). The trigger for social identification, therefore, is individuals' need for self-enhancement, for which purpose they assign themselves to well regarded, attractive and distinctive social groups (Terry and Hogg, 2001).

As discussed earlier, organizational identification allows employees to develop a strong, enduring relationship (developing a feeling of oneness) with their organization (Ashforth and Mael, 1989). Dutton et al. (1994) explain that organizational identification is strongest when (i) the individual's alternative identities are less salient than his/her identity as an organizational member, and (ii) the individual's self-concept and perceived organizational identity have many common attributes. Thus, by investing in CSR activities with the objective of benefiting the community as well as its own employees, an organization enhances employees' identification: they see the organization as being socially responsible and belonging to it meets their own need to enhance their self-esteem. CSR activities also give employees an opportunity to make favorable social comparisons with other organizations, again, in the attempt to improve their self-esteem (Bartel, 2001).

Earlier studies exploring the CSR-organizational identification relationship have not fully explored the mechanism through which the impact of internal and external CSR translates into organizational identification. Both internal and external CSR activities target different stakeholder groups. Based on the group engagement model (Tyler and Blader, 2003), we propose separate mediating mechanisms for internal and external CSR, i.e., perceived internal respect and perceived external prestige, respectively (these are also called status evaluations).

The group engagement model is an appropriate framework because it discusses two types of antecedents of organizational identification (internal and external evaluations), which match our conceptualization of internal and external CSR. Perceived external prestige is individuals' evaluation of their organization's social status (external focus), while perceived internal respect is their evaluation of their own status within the organization (internal focus). The group engagement model suggests that these status evaluations have separate antecedents (Fuller et al., 2006) that are important in gauging employees' relationship with their organization (Tyler and Blader, 2003).

Here, we propose that perceived external CSR contributes to the firm's perceived external prestige whereas perceived internal CSR contributes to employees' perceived internal respect. Further, employees' assessment of CSR activities will vary according to their personal values and work orientation. Employees who see their work as a calling—finding it most meaningful if it has a broader impact or fulfills a greater purpose—will put greater importance on CSR activities of organization. Thus, the current study proposes that the effect of perceived CSR activities on the employees' perception of external prestige and internal respect is moderated by their calling orientation.

### Mediating role of perceived external prestige

Although the literature assumes implicitly that employees' organizational identification and underlying self-enhancement process justifies the way in which CSR affects employee outcomes, most studies have not explored this underlying self-enhancement mechanism. The few exceptions to this (e.g., Jones, 2010) argue that employees satisfy their need for self-esteem by taking pride in belonging to a socially well-regarded organization, which results in favorable attitudes toward the organization. That said, the mediation mechanism that translates the effect of CSR initiatives into favorable attitudes remains unclear (Bhattacharya et al., 2009; Jones, 2010).

The group engagement model provides a sound basis for understanding this psychological mechanism. As discussed above, perceived external prestige and perceived internal respect are two important determinants of employee–organization identification. Perceived external prestige is a commonly used determinant of organizational identification, indicating employees' perception of how outsiders view their organization. Perceived internal respect is a relatively new concept and refers to employees' perception of how their organization treats them.

Perceived external prestige is important because it enhances employees' self-concept and self-worth (Mael and Ashforth, 1992), especially when they believe that outsiders see their organization as being distinctive (Dutton et al., 1994). Proponents of social identity theory (e.g., Van Dick, 2001; Edwards, 2005) argue that individuals prefer being part of prestigious groups because it strengthens their self-esteem. In an organizational context, the firm's external prestige increases its employees' self-esteem as well as their organizational identification (Ashforth and Mael, 1989; Benkhoff, 1997).

Employees identify strongly with companies they perceive as being socially responsible (e.g., Farooq M. et al., 2014; Farooq O. et al., 2014; El Akremi et al., 2015). However, instead of focusing on how external CSR directly affects organizational identification, we argue that such activities are means of enhancing the perceived external prestige of an organization (Kim et al., 2010). According to Pratt (1998), employees will develop a positive social image of their organization if it is involved with a social cause. Community development and philanthropy enhance outsiders' perception of the firm (Fryxell and Jia, 1994; Brammer and Millington, 2005). In particular, external CSR activities reflect characteristics that society tends to recognize and value. This is expected to induce the perception of external prestige, which people evaluate through visible, recognizable symbols, or attributes within that particular society (March and Simon, 1958).

Employees' perception of external prestige stems from external cues such as word of mouth, publicity, and other media (Smidts et al., 2001). Based on these, employees will compare the distinctive, central and enduring practices of their organization with those of other firms when assessing relative prestige (Dutton et al., 1994; Lee et al., 2008). We argue that organizations considered socially responsible have high perceived external prestige in the eyes of their employees, which subsequently affects the latter's organizational identification. Based on the above discussion, we propose the following hypothesis:

***Hypothesis 1:*** Perceived external prestige mediates the relationship between external CSR and organizational identification.

### Mediating role of perceived internal respect

Until the group engagement model (Tyler and Blader, 2003) emerged, the bulk of organizational identification research focused primarily on employees' perception of what others thought of their organization, without differentiating between external prestige and internal respect. Tyler (1999) defines internal respect as individuals' perception that “I am valued by my organization” (p. 219). Being valued thus helps employees to develop a stronger sense of organizational identification insofar as perceived internal respect fulfills their need for self-enhancement (Fuller et al., 2006).

Employees evaluate perceived internal respect through certain cues from the organization, such as its concern for employee health, safety, and well-being, and the extent to which they can participate in decision making. These cues signal to employees that they are central to the organization, and are valued and respected (Tyler and Blader, 2002). Their perception of respect is related to the reputational self and fulfills their need to maintain a positive personal identity (Tyler and Blader, 2002). It is imperative to note that internal respect in this study is evaluated through individuals' “feelings of inclusion or membership in the group and via internal standards of judgment” (Tyler and Blader, 2002, p. 830), also called autonomous judgments. In other words, employees' primary concern is whether they are members in *good standing* and not whether they are in *better standing* than their colleagues (Tyler and Blader, 2002).

Internal CSR initiatives of organization can give employees the necessary cues that it cares about them (Bhattacharya et al., 2008; Rodrigo and Arenas, 2008). We suggest that internal CSR, which focuses on employees' welfare and well-being, sends cues that the organization is benevolent and values its employees. This generates perceived internal respect. This proposition has tangential support from Fuller et al. (2006) who show that human resource practices such as opportunities for extensive training, recognition, and organizational justice are predictors of perceived internal status. Thus, internal CSR initiatives seen to target employees as the beneficiaries have a positive impact on employees' perceived respect and increase their organizational identification. We hypothesize the following:

***Hypothesis 2:*** Perceived internal respect mediates the relationship between internal CSR and organizational identification.

### Moderating role of employees' calling orientation

The literature argues that individual perceptions are affected by different stimuli, which applies equally to perceived CSR (c.f. Glavas and Godwin, 2013). Studies suggest that employees have varying work orientations (c.f. Wrzesniewski et al., 1997; Glavas and Godwin, 2013, p. 20). Some see their work as merely a job or means of getting paid, others may see it in terms of good career prospects and a way to express themselves (e.g., by seeking promotion), and still others might see work as giving their lives meaning or a greater purpose. Some employees might be driven by different combinations of all three (Wrzesniewski, 2003; Wrzesniewski et al., 2003), although for few calling orientation may well be the most important (Glavas and Godwin, 2013).

The multidimensional concept of employees' calling orientation often includes workplace spirituality (Clark et al., 2007), identity (Britt et al., 2001), intrinsic work orientation, and work values (Roberson, 1990). Including these components in calling orientation pushes it closer to a moral perspective, i.e., deontic justice theory, which suggests that people are concerned about justice because unfair treatment violates ethical and moral norms (Folger, 1998, 2001). This perspective also suggests that people react to first-party justice (the treatment they receive—internal CSR) as well as to third-party justice (the treatment of others—external CSR) (Skarlicki and Kulik, 2005). In this context, “others” denotes coworkers (Skarlicki et al., 1998). However, researchers argue that this phenomenon can be extended to CSR targeting external stakeholders (Rupp et al., 2006). In addition, Rupp et al. (2013a, p. 899) argue that employees are likely to see the positive effects of CSR only “if social responsibility was something that the employee valued a priori and thus a deontic or morality-based value structure is still implied.”

Building on these arguments, employees with a higher level of calling orientation are more likely to see their job as a means of achieving a greater purpose due to the firm's perceived external CSR activities (Besharov, 2008) (third-party justice). In other words, the perceived external CSR persuades these employees that their firm has a deontic perspective because it is attempting to meet moral and ethical norms. Based on their inclination toward calling orientation, employees perceive that outsiders give greater weight to external CSR activities and consider their organization to be socially prestigious. This ultimately develops into high perceived external prestige. Thus, employees with a higher calling orientation are likely to have a biased assessment of how important external CSR is to others (Rosso et al., 2010).

Similarly, employees with a higher level of calling orientation are likely to be biased when gauging how important internal CSR (first-party justice) is to them. Organizations have a moral or ethical obligation to help workers feel that their work is meaningful (Michaelson, 2005); membership of an organization with a deontic perspective of justice gives their work positive meaning. The organization's perceived internal CSR activities imply that it is fulfilling moral and ethical norms vis-à-vis its internal stakeholders. In response, these employees are more likely to perceive stronger impact of internal CSR on internal respect than employees with a lower calling orientation.

**Figure 1:**
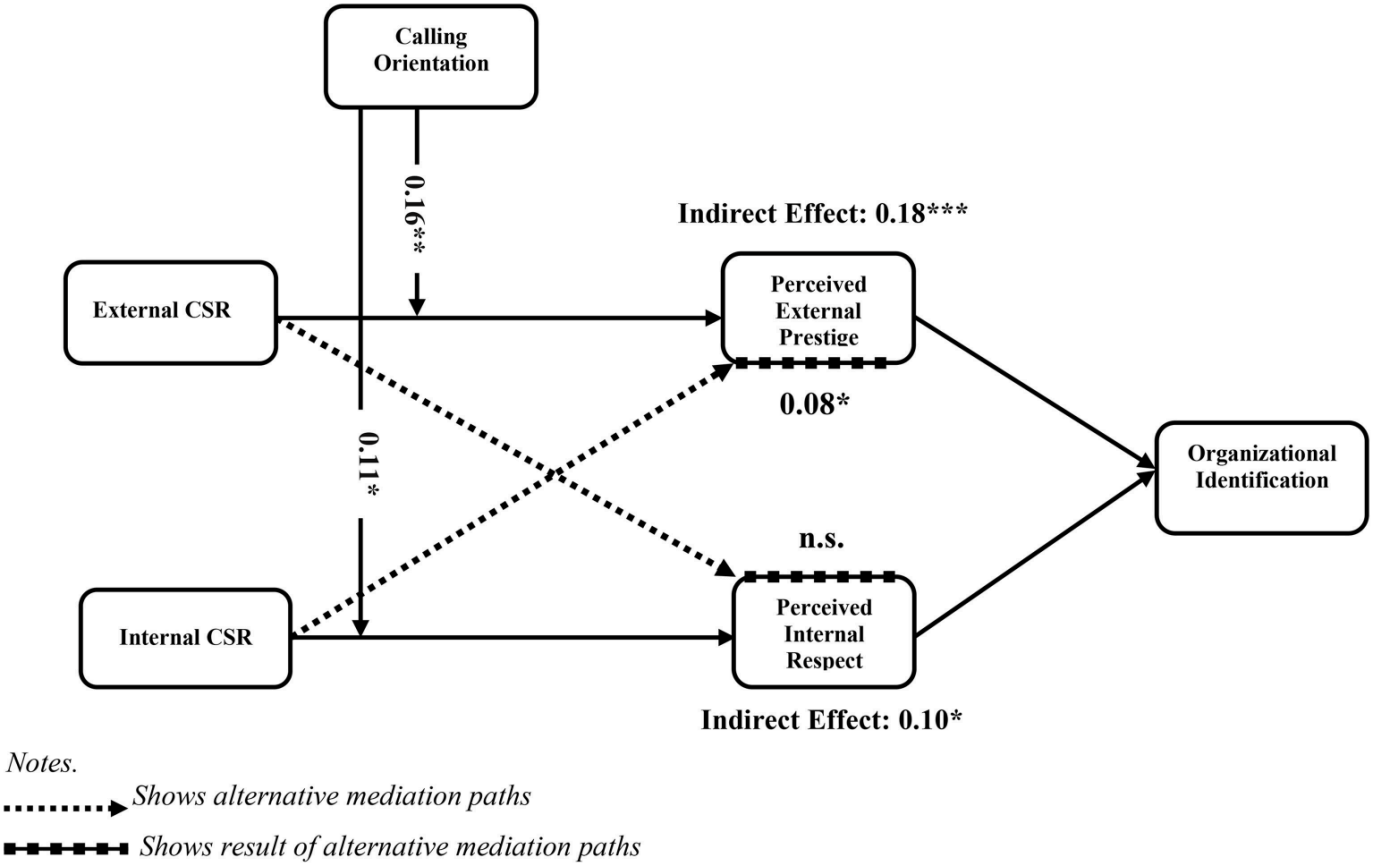
**Hypothesized model**.

Taken together, employees with a higher calling orientation are expected to care more about their firm's CSR initiatives and will be more affected by perceptions of internal and external CSR as they relate to internal/external image (Rosso et al., 2010; Glavas and Godwin, 2013). Hence, the mediating relationships proposed in hypotheses 1 and 2 are conditional and the strength of the relationship depends on employees' calling orientation (see Figure 1). We put forward the following:

***Hypothesis 3:*** The relationship between external CSR and perceived external prestige is moderated by employees' calling orientation such that the stronger the calling orientation, the stronger will be the relationship and vice versa.

***Hypothesis 4:*** The relationship between internal CSR and perceived internal respect is moderated by employees' calling orientation such that the stronger the calling orientation, the stronger will be the relationship and vice versa.

## Methods

### Procedure and sample

A cross-sectional survey (self-administered questionnaire) was developed to collect data for the study. Several members of the Securities and Exchange Commission of Pakistan were interviewed in order to identify which firms the survey should include. Based on these interviews, we selected five multinational corporations that run vigorous, high-profile CSR campaigns, making them visible to the public (external stakeholders). We used interviews for this purpose because there is no published data available on Pakistan in the context of this study. Having asked each firm's human resources department for permission to administer the survey, we sent them a copy of the questionnaire (in English), accompanied by a cover letter explaining the purpose of the study and assuring all respondents they would remain anonymous. We also ensured that the questionnaire was administered without any direct involvement by the human resources department.

We focused on banking and telecommunication organizations, specifically on companies engaged in multiple CSR initiatives for the local community. For instance, one large multinational corporation participating in this survey has provided services and contributed relief funds in response to natural and manmade disasters such as the Awaraan earthquake, the internally displaced persons crisis and the famine in Tharparkar. Other companies have provided drinking water filtration and sanitation facilities, built and equipped IT labs at educational institutions, and introduced online teaching-learning content and e-learning to improve the quality of education.

A total of 550 questionnaires were distributed, of which 430 were returned and 414 analyzed. All the respondents were Pakistani citizens and, on average, 33 years old (SD = 9.12). The majority were male (85%), similar to several other studies in the field (e.g., Kim et al., 2010). Most respondents (66%) had at least a Master's degree and had worked at the organization for 7 years, on average (SD = 7.14). Respondents held a variety of positions, including assistant manager (48.2%), manager (21.4%), and technical officer (19.5%).

### Measures

All measures—except the control variables—were measured on a five-point Likert scale ranging from “strongly disagree” (1) to “strongly agree” (5). The five-item scale adapted from Mael and Ashforth (1992) was used to measure perceived external prestige. The sample item was “People in my community think highly of my organization.” The six-item scale used by Blader and Tyler (2009), based on Tyler et al. (1996), was used to measure perceived internal respect, where the sample item was “Managers think that I have valuable insights and ideas.” Organizational identification was measured on a five-item scale used by Blader and Tyler (2009), adapted from Mael and Ashforth (1992), where the sample item was “When I talk about the organization, I usually say ‘we’ rather than ‘they’.”

Perceived internal and external CSR were measured on a 12-item scale adapted from (Turker, 2009b) 17-item CSR scale, which measures four dimensions^1^. Five of the 12 items measured internal CSR (i.e., CSR to employees), where the sample item was “Our company supports employees who want to acquire additional education.” The remaining seven items measured external CSR (i.e., CSR to social and nonsocial stakeholders), where the sample item was “Our company contributes to campaigns and projects that promote the well-being of the society.” Finally, employees' calling orientation was measured on a three-item scale from Steger et al. (2012), where the original sample item was “I know my work makes a positive difference in the world.”

Control variables—in this case, age, gender and experience—were included in survey to rule out other possible explanations for any significant relationships. Variables such as age and gender can have a significant impact on organizational identification at the individual level (Riketta, 2005).

### Data analysis

The data was analyzed and the hypotheses tested using SPSS 21 and AMOS 21. The data screening stage incorporated missing value analysis, multivariate outliers, normality, descriptive statistics, multicollinearity, homoscedasticity and correlation analyses. Table 1 summarizes the descriptive statistics, indicating moderate correlation among the variables. None of the control variables are significantly correlated with any of the dependent variables. Petersitzke (2009) suggests using only the control variables significantly correlated with the dependent variable because using non-significant terms can affect the coefficient values for significant terms in regression model. Accordingly, we have not included the control variables in the final analysis.

**Table 1:** **Descriptive statistics**.

**Variable**	**Mean**	**SD**	**1**	**2**	**3**	**4**	**5**	**6**	**7**	**8**
1. Gender^a^	1.15	0.36								
2. Age	33.20	9.12	−0.19^**^							
3. Experience^b^	7.10	7.14	−0.07	0.78^**^						
4. Internal CSR	3.62	0.69	−0.10	0.11^*^	0.07					
5. Internal respect	3.89	0.62	−0.07	0.04	0.04	0.44^**^				
6. Calling orientation	3.94	0.60	−0.01	0.08	0.80	0.32^**^	0.64^**^			
7. External CSR	3.91	0.61	−0.01	0.21^**^	0.12^*^	0.53^**^	0.37^**^	0.27^**^		
8. External prestige	4.17	0.54	0.03	0.08	0.09	0.46^**^	0.48^**^	0.53^**^	0.40^**^	
9. Organizational identification	4.14	0.61	−0.00	0.04	0.03	0.30^**^	0.36^**^	0.31^**^	0.30^**^	0.43^**^

N = 414;

* p < 0.05;

** *p < 0.01*.

a *1 = male; 2 = female*.

b *Years of work experience*.

Construct validity was assessed through confirmatory factor analysis using AMOS 21. In order to assess the model's goodness of fit, we use the following indices (Byrne, 2001): the comparative fit index (CFI), the Tucker–Lewis index (TLI), CMIN/df and the root-mean square error of approximation (RMSEA). According to Hair et al. (2010) and Kline (2011), a good model fit should have CFI and TLI values above 0.90 and a RMSEA score below 0.08. The results of the first model (applied to all items of six factors) show relatively poor fit statistics (CMIN/df = 2.29, CFI = 0.89, TLI = 0.88, RMSEA = 0.05). The second model was tested after removing four low-factor loading items (one for calling orientation, one for internal respect and two for external CSR)^2^. In this case, the model fit statistics improve significantly, reaching acceptable levels (CMIN/df = 1.43, CFI = 0.97, TLI = 0.97, RMSEA = 0.03).

Two additional models (Bentler and Bonett, 1980) are analyzed to assess the appropriateness of the proposed measurement model. The first alternative model is tested by loading all the items on a single factor. The results indicate a poor fit (CMIN/df = 6.72, CFI = 0.77, TLI = 0.62, RMSEA = 0.18). In the second alternative model, we introduce a four-factor solution in which the two status evaluations are merged into one, and internal and external CSR are combined. These results also indicate a poor fit to the data (CMIN/df = 5.82, CFI = 0.79, TLI = 0.74, RMSEA = 0.11). Thus, the results of the six-factor model show a superior fit^3^ compared to the alternative models.

We follow Hair et al. (2010) in measuring the discriminant and convergent validities of all the scales. To establish convergent validity, the AVE > 0.50; to establish reliability, the CR > 0.70; and to establish discriminant validity, MSV < AVE and ASV < AVE. Table 2 shows that all the measures used are reliable and valid and meet these criteria. The exceptions are perceived internal CSR and perceived external CSR, which show low convergent validity.

**Table 2:** **Reliability and validity**.

**Variable**	**CR**	**AVE**	**MSV**	**ASV**
Internal CSR	0.81	0.46	0.45	0.30
Organizational identification	0.87	0.58	0.24	0.16
Perceived internal respect	0.86	0.56	0.32	0.26
Perceived external prestige	0.85	0.52	0.32	0.26
External CSR	0.80	0.45	0.44	0.27
calling orientation	0.70	0.54	0.37	0.26

*N = 414; CR, Composite Reliability; AVE, Average variance extracted; MSV, Maximum Shared Variance; ASV, Average Shared variance*.

The common latent factor test is conducted in structural equation modeling to gauge the common method variance of the data. This is more robust than the commonly used Harman's single-factor test. The results reveal a shared variance of 22% among all items, implying that the data has no major common method variance issue.

## Results

The structural regression model is tested using AMOS 21; the multiple regression analysis employs SPSS 21. The results of structural regression model show a good fit to the data (CMIN/df = 1.65; RMSEA = 0.04, TLI = 0.96, CFI = 0.96). Hypothesis 1 concerns the mediating effect of perceived external prestige between external CSR and organizational identification. However, the model is characterized by multi-mediation, which AMOS 21 cannot test directly. For this purpose, we use the phantom model technique (Macho and Ledermann, 2011) along with 5000 bootstrapping samples (Preacher and Hayes, 2008), which enables us to determine the specific indirect effects and their significance levels.

The results of structural regression model show that external CSR has a positive relationship with perceived external prestige (unstandardized estimate = 0.51, SE = 0.07, *p* < 0.001) and perceived external prestige has a positive impact on organizational identification (unstandardized estimate = 0.36, SE = 0.08, *p* < 0.001). The results also indicate that external CSR does not have a significant effect on organizational identification (unstandardized estimate = 0.13, SE = 0.10, *p* > 0.05). The phantom model technique reveals that external CSR has a significant and positive indirect effect on organizational identification through perceived external prestige (unstandardized estimate = 0.18, SE = 0.07, *p* < 0.001). The results show that perceived external prestige fully mediates the relationship between external CSR and organizational identification as the direct effect of external CSR on organizational identification is insignificant, thus supporting Hypothesis 1 (see Table 3).

**Table 3:** **Mediation analysis results**.

	**Point of estimate**	**S.E**	**BC 95% CI**	
			**Lower**	**Upper**
Total effect of external CSR	0.31^***^	0.13	0.11	0.63
Direct effect of external CSR	0.13	0.12	−0.09	0.38
Indirect effect (via perceived external prestige)	0.18^***^	0.07	0.08	0.35
Total effect of internal CSR	0.08	0.11	−0.14	0.29
Direct effect of internal CSR	−0.02	0.11	−0.25	0.20
Indirect effect (via perceived internal respect)	0.10^*^	0.05	0.02	0.22

*BC, Biased Corrected (5000 bootstrapping samples)*.

* p < 0.05;

*** *p < 0.001*.

Hypothesis 2 concerns the mediating effect of perceived internal respect between internal CSR and organizational identification. Using the same method outlined above for Hypothesis 1, the results of structural regression model show that internal CSR has a positive relationship with perceived internal respect (unstandardized estimate = 0.51, SE = 0.07, *p* < 0.001) and perceived internal respect has a positive effect on organizational identification (unstandardized estimate = 0.20, SE = 0.08, *p* < 0.01). Internal CSR does not have a significant effect on organizational identification (unstandardized estimate = −0.02, SE = 0.11, *p* > 0.05). The phantom model technique shows that internal CSR has a significant and positive indirect effect on organizational identification through perceived internal respect (unstandardized estimate = 0.10, SE = 0.05, *p* < 0.05). The results show that perceived internal respect fully mediates the relationship between internal CSR and organizational identification as the direct effect of internal CSR on organizational identification is insignificant. These results provide support for Hypothesis 2 (see Table 3).

In *post hoc* analysis, we simultaneously test the path from external CSR to organizational identification via perceived internal respect, and from internal CSR to organizational identification via perceived external prestige. The results reveal that external CSR affects organizational identification via prestige, whereas internal CSR affects organizational identification via both mechanisms, i.e., prestige and respect (unstandardized estimate = 0.08, SE = 0.06, *p* < 0.01). Although we have not hypothesized these relationships in the study, this result offers some interesting insight into the impact of internal CSR: while internal CSR does not affect organizational identification directly, it does affect employee identification indirectly via prestige and respect (this path is shown by the dotted line in Figure 1).

In order to test hypotheses 3 and 4, we use the recently developed PROCESS macro for SPSS (Hayes, 2013) with 5000 bootstrap samples as recommended by (MacKinnon et al., 2012). This macro is both useful and appropriate for calculating the interaction effects (Hayes, 2013). Hypothesis 3 states that employees' calling orientation moderates the positive relationship between external CSR and perceived external prestige, such that the higher the calling orientation, the stronger will be the relationship, and vice versa. Table 4 shows that the interaction term (external CSR × calling orientation) has a significant effect on perceived external prestige. This implies that the effect of external CSR on perceived external prestige increases in tandem with calling orientation. The results, therefore, support the Hypothesis.

**Table 4:** **Moderation hypotheses results**.

	**Hypothesis 3 Perceived External Prestige**				**Hypothesis 4 Perceived Internal Respect**			
	**Point of estimate**	***S.E***	**BC 95% CI**		**Point of estimate**	***S.E***	**BC 95% CI**	
			**Lower**	**Upper**			**Lower**	**Upper**
External CSR	0.25^***^	0.04	0.18	0.32				
Calling orientation	0.40^***^	0.04	0.33	0.47				
External CSR × calling orientation	0.16^**^	0.05	0.26	0.05				
Internal CSR					0.23^***^	0.03	0.16	0.30
Calling orientation					0.57^***^	0.04	0.49	0.64
Internal CSR × calling orientation					0.11^*^	0.05	0.01	0.21

BC, Biased Corrected (5000 bootstrapping samples); N = 414;

* p < 0.05;

** p < 0.01;

*** *p < 0.001*.

Hypothesis 4 states that calling orientation moderates the positive relationship between internal CSR and perceived internal respect, such that the higher the calling orientation, the stronger will be the relationship, and vice versa. Table 4 shows that the interaction term (internal CSR × calling orientation) has a significant effect on perceived internal respect. The effect of internal CSR on perceived internal respect increases with higher levels of calling orientation. These results also support the Hypothesis.

**Figure 2:**
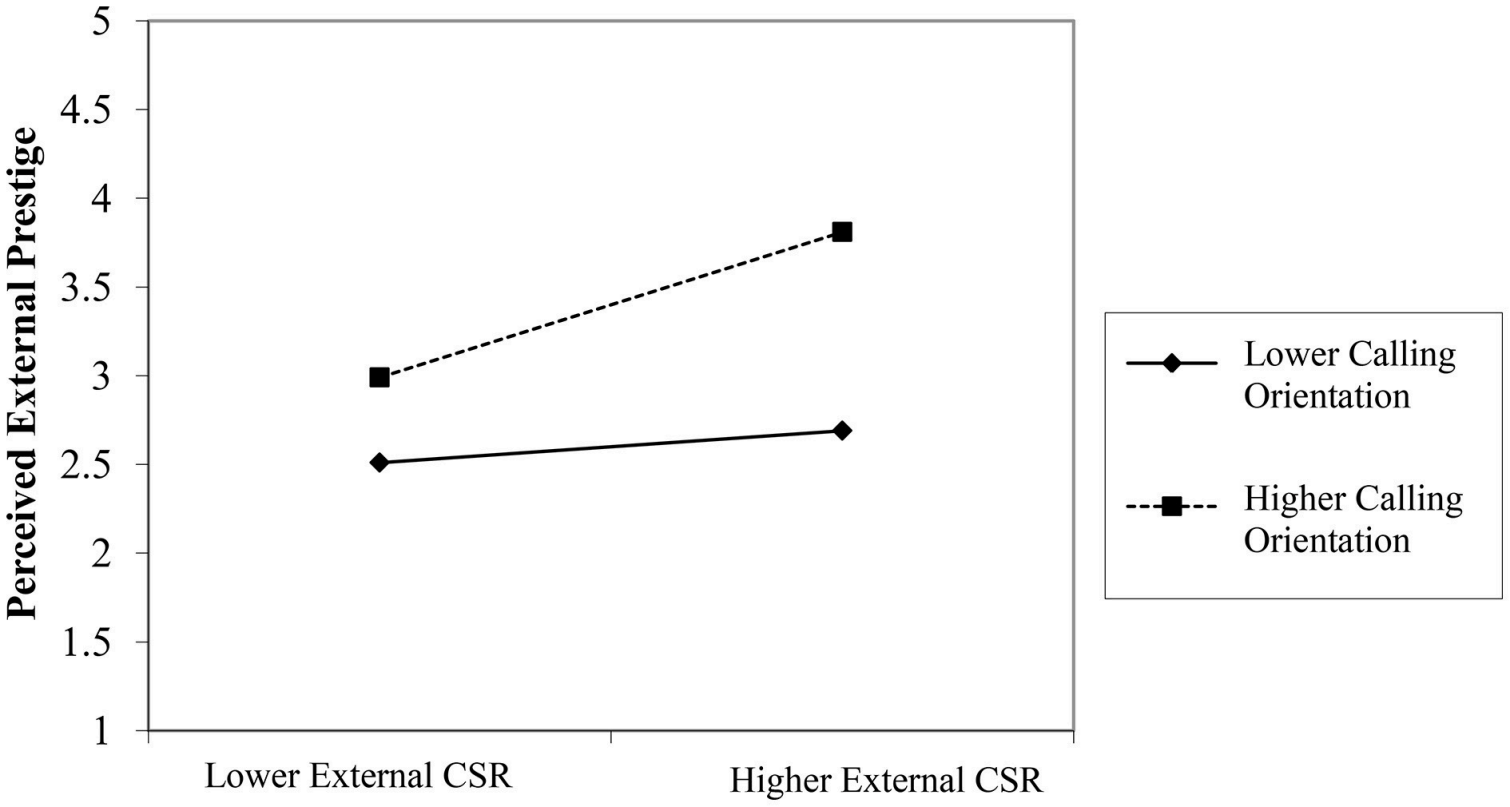
**Hypothesis 3**.

**Figure 3:**
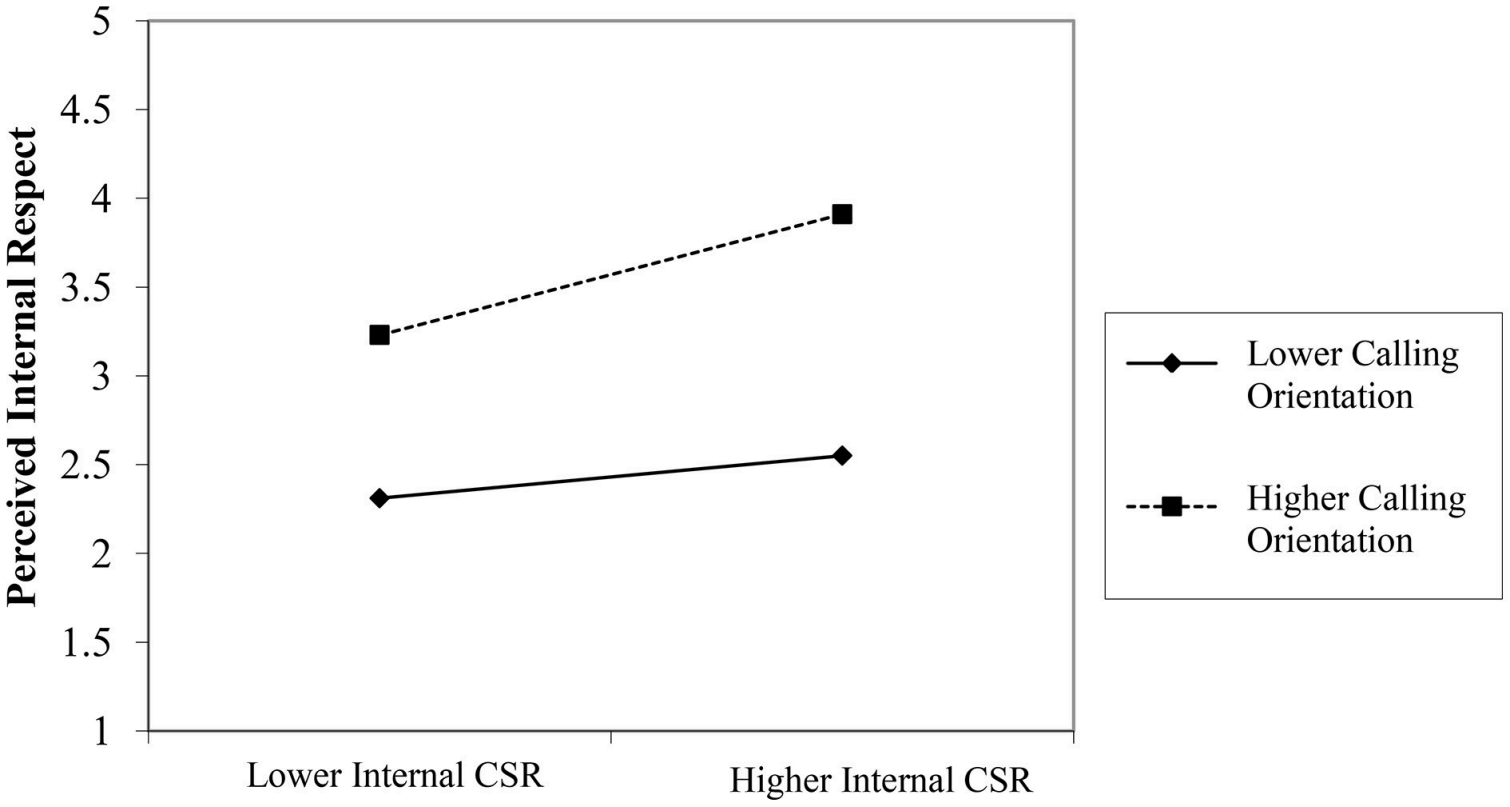
**Hypothesis 4**.

Figures 2, 3 illustrate these relationships. The results of the simple slope test in Table 5 show that the impact of perceived external CSR on perceived external prestige varies significantly at lower and higher levels of calling orientation. At a lower calling orientation, the unstandardized estimate is 0.15 with *p* < 0.01, whereas at a higher calling orientation, the unstandardized estimate is 0.34 with *p* < 0.001. Similarly, in the case of perceived internal respect, the effect of perceived internal CSR at a lower calling orientation yields an unstandardized estimate of 0.16 with *p* < 0.001. At a higher calling orientation, the unstandardized estimate is 0.29 with *p* < 0.001.

**Table 5:** **Simple slope test**.

**Independent variable**	**Dependent variable**	**Effect**	
		**At lower calling orientation**	**At higher calling orientation**
External CSR	Perceived external prestige	0.15^**^	0.34^***^
Internal CSR	Perceived internal respect	0.16^***^	0.29^***^

N = 414;

** p < 0.01;

*** *p < 0.001*.

## Discussion

This study has explored the mechanisms through which perceived internal and external CSR affect the extent to which employees identify with their organization. We have also examined how employees' calling orientation moderates the relationship between CSR perceptions and two different types of status evaluations, i.e., perceived external prestige and perceived internal respect. While previous research has established the relationship between CSR perceptions and organizational identification (e.g., Rodrigo and Arenas, 2008), the underlying processes are not well understood. This study contributes to the literature by exploring the different pathways through which external and internal CSR build employees' identification.

### Mediation of perceived external prestige between external CSR and identification

The results of the empirical analysis reveal that perceived external prestige fully mediates the relationship between perceived external CSR and organizational identification. The direct effect of perceived external CSR on organizational identification becomes insignificant in the presence of perceived external prestige. The perception of external CSR affects perceived external prestige, which subsequently has a positive impact on organizational identification.

These results extend previous findings on the direct effect of CSR on identification (Collier and Esteban, 2007; Rodrigo and Arenas, 2008; Farooq O. et al., 2014) by showing how this effect occurs. The findings also indicate that employees are concerned with organizational activities that support external stakeholders, which they see as an important part of developing a positive social image (Rego et al., 2010). According to social identity theory, the aim of enhancing one's self-esteem is achieved by members of a group (the organization) if that group is considered highly prestigious by the out-group (in this case, society). This aim compels employees to identify with their organization (Ashforth and Mael, 1989). Thus, it is through perceived external prestige that the effects of external CSR translate into employees' identification with their organization.

### Mediation of perceived internal respect between internal CSR and identification

The results support the hypothesis that perceived internal respect mediates the relationship between perceived internal CSR and organizational identification. In the presence of perceived internal respect, the effect of internal CSR on organizational identification is insignificant. This implies that, if employees are treated well in the workplace—in the form of training opportunities, respect for human rights, work-life balance, health and safety—then this is likely to enhance their self-image. Consequently, internal CSR compels employees to identify with the organization (Tyler and Blader, 2003). Our data analysis supports the theoretical rationale for using the group engagement model to enhance our understanding of the psychological processes underlying the relationship between CSR and organizational identification.

Another important insight concerns the insignificant total effect of internal CSR on organizational identification. The corresponding *post hoc* analysis highlights that perceived internal CSR does not contribute directly to employees' organizational identification. Farooq O. et al. (2014) give a possible explanation for this, suggesting that, overall, companies in developing countries carry out internal CSR on a far smaller scale. Therefore, it may not have a direct impact on employees' identification. However, internal CSR indirectly influences employee identification via perceived internal respect and perceived external prestige as demonstrated through *post hoc* analysis. This offers an interesting avenue for future research.

### Moderating role of employees' calling orientation

Hypotheses 3 and 4 concern the moderating effect of calling orientation on the relationship between external CSR and perceived external prestige, and between internal CSR and perceived internal respect. Our analyses support these hypotheses, showing that organizational CSR activities have a varying degree of influence over different employees, depending on the importance they assign to CSR. In other words, an employee who puts high value on CSR is more likely to find that the organization's CSR activities enhance his or her self-esteem (Glavas and Godwin, 2013).

### Theoretical contributions

According to Jones (2010) and He and Brown (2013, p. 19) there is dearth of research regarding how CSR affects employee attitudes and behaviors—especially employees' organizational identification. Furthermore, CSR has occupied a central position in strategic management and consumer research whereas there has been meager contribution regarding how CSR affects employees' organizational identification in organizational behavior literature (He and Brown, 2013). The past research in this realm has observed that employees' perceptions about the status and identity of the organization can influence their level of organizational identification (Tyler and Blader, 2003; Blader and Tyler, 2009). Consequently, organizational identification can be related to the emerging agenda of CSR as postulated by Glavas and Godwin (2013) and He and Brown (2013). In the backdrop of this recent theoretical development, this study has allowed us to make a substantial contribution to delineate both mediating and moderating mechanisms which actually facilitate the relationship between CSR and employees' organizational identification. This contribution is vital in the sense that it allows us to reveal the missing linchpins in this apparent direct relationship. In so doing, we make two vital theoretical contributions.

First, we underline the mediating effect of status evaluations through the group engagement model (Tyler and Blader, 2003). This mediated model enhances our theoretical understanding of this important relationship and explains how CSR activities help develop employees' organizational identification. Thus, the study delineates the nature of the relationship between CSR and employees' identification, provides an improved understanding of the processes at work, and highlights the implications for managing CSR initiatives in organizations. For instance, our findings suggest that future studies should differentiate between external and internal CSR initiatives when examining their impact on employee outcomes. While, importantly, both external and internal CSR activities follow separate psychological processes, the *post hoc* analysis suggests that perceived external prestige serves as a mediating mechanism for both types of CSR.

Finally, the study contributes to the literature by conceptualizing and testing employees' calling orientation (Walsh et al., 2003) as a moderating mechanism to explicate the alleged direct relationship between CSR and employee identification. We show how CSR has a different impact across the organization's employees and that this impact depends on employee characteristics: not all employees will respond equally positively. Micro CSR researchers need to take this boundary condition into account to understand the phenomenon better.

### Practical implications

The study's findings have several implications for managers when formulating and implementing CSR strategies. The first concerns the importance of both internal and external CSR and the associated payoff for the organization. Employee attitudes and behaviors are scarce, intangible and unique resources with no perfect substitute (Ballou et al., 2003; Fulmer et al., 2003). Therefore, CSR helps maintain an effective workforce, creating a competitive advantage that affects business performance (Branco and Rodrigues, 2006).

The results demonstrate that the benefits of CSR activities are not limited to external prestige and external stakeholders, but also help in changing the attitudes of internal stakeholders. We also suggest that both types of CSR are effective in strengthening employees' identification with their organization. Managers should help employees understand perceived external prestige by highlighting the positive impact of CSR activities.

Finally, the findings suggest that CSR does not only have a positive impact on employees' identification, but it also helps the firm boost its perceived external prestige. This is important to take into account when formulating effective internal strategies to influence employees' related attitudes and behaviors. The moderated mediation model shows that the impact of CSR on employees' identification varies from individual to individual depending on their calling orientation. Managers should keep in mind these individual differences when gauging the role of CSR in this context. Thus, there is no “best way” of carrying out CSR and the difference in employees' calling orientation plays an important role in evaluating how effective a particular initiative is likely to be.

### Limitations and directions for future research

The study has several limitations. First, the sample does not fully represent the population at large, given that it comprises only multinational corporations engaged in CSR activities. This may restrict the range of the external CSR measure and limit the extent to which we can generalize the results across other organizations.

Second, all the measures in this study draw on self-reported data on individuals' perceptions and attitudes. Although researchers argue that this is a useful and valid source of data (Glick et al., 1986; Spector, 1994), it can also create common method bias (Spector, 1994). We have followed various recommendations for minimizing this bias, such as ensuring the confidentiality of respondents, providing a cover letter that explains the purpose of the study, and measuring predictors and dependent variables separately (Podsakoff et al., 2003). Since all the constructs were measured using a cross-sectional design, a single-factor model was also analyzed. This showed a poor fit to the data, implying that no single factor explains the bulk of the variance. Thus, common method variance was not a serious concern in our dataset.

In future, we suggest using a longitudinal design to address any concerns relating to causal relationships and cross-sectional data. The use of a more sophisticated design will provide robust evidence of this mediating relationship (Aguinis and Glavas, 2012). We also propose including the behavioral outcomes of organizational identification (e.g., task and contextual performance, actual turnover), which can be measured using objective data. This would strengthen the research design and enhance the reliability of the results.

Another important avenue for future research may be related to conceptualization and operationalization of perceived internal respect with slightly different lens. In present study conceptualization of perceived internal respect only captured the employees' perceptions about how they are treated within organization (self-focused), which ignores the collective/generalized side of respect. In other words, when an employee is asked to report the internal respect related to all employees or colleagues i.e., in “we or they” mode instead of reporting in “I” mode (please refer to perceived internal respect scale in Supplementary Material Appendix I), then s/he may report differently. This is important from our point of view because perceived external prestige is about a collective entity, and by using this proposed conceptualization we will be able to capture collective side of internal respect which will provide us an opportunity to explore the competing roles of these mediating mechanisms.

Finally, a potential line of research is that of other boundary conditions, such as social culture, personality traits, and other contextual factors, which would help improve our understanding of the mediating mechanism. This, in turn, would help managers better understand the varying effect of CSR activities on different employees.

## Author contributions

IH helped designing the study, and wrote the manuscript. ZR helped designing and writing the manuscript. GA collected and analyzed the data and provided feedback on the manuscript. OF repositioned and fine-tuned the paper, wrote introduction, and provided feedback on the manuscript.

### Conflict of interest statement

The authors declare that the research was conducted in the absence of any commercial or financial relationships that could be construed as a potential conflict of interest.

## Footnotes

^1^We used items related to two dimensions which were CSR towards social and nonsocial stakeholders, and CSR toward employees. As per the conceptualization of CSR i.e., CSR is a set of initiatives of an organization that go beyond the notion of profit-making or compliance with the law (McGuire, 1963; Davis, 1973; McWilliams et al., 2006; Aguilera et al., 2007; De Roeck et al., 2014), we have excluded CSR directed toward customers and government.

^2^The removed items were as follows: Perceived external CSR (i) “Our company encourages its employees to participate in voluntary activities” and (ii) “Our company supports nongovernmental organizations working in problematic areas.” Perceived internal respect (i) “Managers think it would be difficult to replace me.” Calling orientation (i) “My work really makes no difference to the world.”

^3^The chi-square test of difference was not applied because the model's superiority was evident from the fit indices.

## Supplementary material

The Appendix for this article can be found online at: http://journal.frontiersin.org/article/10.3389/fpsyg.2016.00788
