# Supplementary material for: How Do Internal and External CSR Affect Employees' Organizational Identification? A Perspective from the Group Engagement Model
Source: Front Psychol. 2016 May 30;7:788. doi: 10.3389/fpsyg.2016.00788 (PMC4884747; doi:10.3389/fpsyg.2016.00788)
Supplement: Supplementary file 1 [file Appendix.docx]

**Appendix I: Measurement Scales**

**Organizational Identification**

1. When someone praises the organization, it feels like a personal compliment.
2. When someone criticizes the organization, it feels like a personal insult.
3. When I talk about the organization, I usually say “we” rather than “they.”
4. The organization’s successes are my successes.
5. If a story in the media criticized the organization, I would feel embarrassed.

**Perceived External Prestige**

1. People in my community think highly of my organization.
2. It is considered prestigious in the community to be an employee of this organization.
3. My company is one of the best companies in its field.
4. People are impressed when I tell them where I work.
5. My organization does not have a good reputation in my community. (R)

**Perceived Internal Respect**

1. Managers respect the work I do.
2. Managers respect my work-related ideas.
3. Managers think highly of the quality of my work.
4. Managers appreciate my unique contributions on the job.
5. Managers think that I have valuable insights and ideas.
6. Managers think it would be difficult to replace me.

**Calling Orientation**

1. My work really makes no difference to the world. (R)
2. The work I do serves a greater purpose.
3. I know my work makes a positive difference in the world.

**Perceived Internal CSR**

1. Our company supports employees who want to acquire additional education.
2. Our company policies encourage the employees to develop their skills and careers.
3. Our company implements flexible policies to provide a good work and life balance for its employees.
4. The management of our company is primarily concerned with employees’ needs and wants.
5. The managerial decisions related with the employees are usually fair.

**Perceived External CSR**

1. Our company contributes to campaigns and projects that promote the well-being of the society.
2. Our company implements special programs to minimize its negative impact on the natural environment.
3. Our company participates in activities which aim to protect and improve the quality of the natural environment.
4. Our company encourages its employees to participate in voluntary activities.
5. Our company supports nongovernmental organizations working in problematic areas.
6. Our company targets sustainable growth which considers future generations.
7. Our company makes investment to create a better life for future generations.
